# Challenges in Antifungal Therapy in Diabetes Mellitus

**DOI:** 10.3390/jcm9092878

**Published:** 2020-09-06

**Authors:** Sazlyna Mohd Sazlly Lim, Mahipal Sinnollareddy, Fekade Bruck Sime

**Affiliations:** 1 Centre for Translational Anti-Infective Pharmacodynamics, School of Pharmacy, University of Queensland, Brisbane 4102, Australia; s.mohdsazllylim@uq.net.au; 2 Department of Medicine, Faculty of Medicine and Health Sciences, Universiti Putra Malaysia, Seri Kembangan 43400, Malaysia; 3 Therapeutic Goods Administration, Canberra 2609, Australia; mahi716@gmail.com; 4 UQ Centre for Clinical Research, Faculty of Medicine, University of Queensland, Brisbane 4029, Australia

**Keywords:** *Candida*, biofilms, diabetes, candidiasis, pharmacokinetic, pharmacodynamic

## Abstract

Diabetic patients have an increased propensity to *Candida* sp. infections due to disease-related immunosuppression and various other physiological alterations. The incidence of candidiasis has increased in number over the years and is linked to significant morbidity and mortality in critically ill and immunosuppressed patients. Treatment of infection in diabetic patients may be complicated due to the various disease-related changes to the pharmacokinetics and pharmacodynamics (PK/PD) of a drug, including antifungal agents. Application of PK/PD principles may be a sensible option to optimise antifungal dosing regimens in this group of patients. Further studies on PK/PD of antifungals in patients with diabetes mellitus are needed as current data is limited or unavailable.

## 1. Introduction

We read with great interest the review by Rodrigues et al. which described the epidemiology and pathophysiology of *Candida* sp. infection in patients with diabetes mellitus (DM) [1]. Diabetic patients are more susceptible to infections due to the hyperglycemic environment that promotes (1) immune dysfunction, such as damage to the neutrophil function, suppression of the antioxidant complex, and humoral immunity; (2) micro- and macro-angiopathies; (3) decrease in the germicidal activity of urine; (4) gastrointestinal and urinary motility dysfunction; (5) quantity of medical procedures in these patients [2].

*Candida* sp. such as *C. albicans*, *C. glabrata*, *C. tropicalis*, *C. parapsilosis*, and *C. krusei* cause opportunistic infections [3]. However, in individuals who are immunocompromised, such as patients with DM, these species can cause invasive infections. Numerous studies have shown the link between *Candida* sp. infection and DM [4,5,6,7,8]. One study reported a higher risk (4.4 times) of developing oral candidiasis in elderly patients with DM when compared with those without DM [4]. Several other studies described DM as one of the risk factors for vulvovaginal candidiasis in their cohorts [5,6]. Furthermore, a prospective case-control study reported DM in 62.5% of patients with *Candida* bloodstream infection [8].

High mortality rates have been reported for patients with *Candida* sp. infection, particularly in those with *Candida* sp. bloodstream infection. Barchiesi et al. [9] for example, reported a mortality rate of 28–45% in patients with candidaemia. Similarly, in an earlier study of candidaemia in immunocompromised patients, the mortality rates associated with *C. krusei* and *C. albicans* were 49% and 28%, respectively [10]. Therefore, adequate treatment is of tremendous importance in this group of patients. However, treatment of infection in a diabetic patient is not as straight forward as those without DM, as we will discuss going forward.

## 2. Effect of Diabetes Mellitus on the Pharmacokinetics of Antifungal

It has been perceived that the pharmacokinetics (PK) and pharmacodynamics (PD) of drugs are altered in patients with DM [11]. DM may affect the pharmacokinetics of numerous drugs, including antifungals, by affecting absorption of the drug, either due to delayed gastric emptying or slower absorption. The prevalence of delayed gastric emptying in diabetic patients has ranged from 28% to 65% [12]. Several studies have demonstrated slower absorption of a drug such as tolazamide [13] and anti-tuberculosis drugs [14] in patients with DM.

DM can also change the distribution of drug via non-enzymatic glycation of albumin [15]. A study by Szkudlarek et al. [15] showed that non-enzymatic glycation of albumin causes structural change to albumin resulting in weaker interactions between ketoprofen and glycated albumin. Reduced drug–protein binding would, in turn, cause an increase in the unbound concentration of the drug, e.g., itraconazole, as shown in a study by Arredondo et al. [16], which looked at protein binding of itraconazole and fluconazole in patients with DM. Changes to the unbound concentration of antifungals are particularly important for antifungals that are highly protein-bound, such as amphotericin B (95–99%) [17], itraconazole (99%) [18], ketoconazole (84%) [19], miconazole (93%) [20], caspofungin (96%) [21], anidulafungin (99%) [18], and micafungin (99%) [18]. Vanstraelen et al. [22] demonstrated a positive relationship between voriconazole plasma protein binding and plasma albumin concentrations in critically ill patients based on a multivariate analysis (*p* < 0.001). This observation indicated that higher unbound voriconazole concentrations is associated with decreasing albumin concentrations. Kurland et al. [23], on the other hand, found that hypoalbuminemia in critically ill patients was associated with higher caspofungin clearance (*r* = −0.31; *p* = 0.062).

DM could also impair drug distribution into tissues due to disease-induced microvascular changes and reduced vascular permeability. Given the incidence and severity of diabetic foot infections and possibility of reduced tissue distribution, antimicrobial tissues penetration in DM patients, particularly in the setting of diabetic foot infections is of interest. The difference in tissue penetration in DM patients appear to be dependent on local blood flow, capillary density, and to some extent on the acute inflamed foot lesions [24,25]. In patients with chronic diabetes and chronic foot infections, drug tissue penetration is bound to be lower than other patients. Although data on antifungal drugs is lacking, studies have demonstrated that antibiotics like vancomycin, fosfomycin, and macrolides (except for telithromycin), which in general distributes well into tissues, appear to have significantly reduced tissue distribution in DM patients. Whereas for antibiotics like oxazolidinones, daptomycin, tigecycline, and fluoroquinolones, the tissue distribution tends to be sufficient and independent of disease state [24,25].

DM is also associated with liver diseases such as non-alcoholic fatty liver disease [26] and diabetic hepatosclerosis [27], causing abnormal hepatic function, which may affect drug biotransformation. The impact of diabetes on drug biotransformation has been observed in animal models [28,29]. Watkins et al. [28] demonstrated in their study that biliary excretion of acetaminophen decreased by 65%, with a 280% increase in urinary excretion, resulting in unaltered total clearance. Antifungals such as itraconazole, voriconazole, and micafungin undergo hepatic metabolism, which would be affected in the setting of hepatic impairment [18]. Changes to the hepatic metabolism of a drug will also affect its serum concentration. Micafungin, for example, exhibited reduced exposure in patients with moderate hepatic impairment (Child–Pugh score 7–9) in comparison with healthy volunteers (mean AUC_0–∞_, 97.5 versus 125.9 µg.h/mL, *p* = 0.03) [30].

## 3. Challenges in the Treatment of *Candida* sp. Infections in Diabetic Patients

### 3.1. Antifungal Resistance

Rodrigues et al. [1] mentioned in their review how hyperglycaemia in diabetic mice reduced the susceptibility of *Candida* sp. to voriconazole and amphotericin B [31]. Reduced susceptibility to antifungals in diabetic patients has also been demonstrated in the clinical setting [32,33,34]. Bhuyan et al. [32] observed a significant difference in the number of *C. albicans* and *C. parapsilosis* that is resistant to fluconazole in diabetes patients compared to healthy controls. Similarly, a higher proportion of patients with DM (47%) were reported to have *Candida* sp. isolates that are resistant against ketoconazole in another study [33]. Furthermore, Al-Attas et al. [34] found that the *Candida* sp. isolated from their diabetic patients had higher rates of resistance to five antifungal drugs (flucytosine, fluconazole, ketoconazole, miconazole, econazole). In contrast, in the healthy controls, none of the isolated yeast showed any resistance to the tested antifungal agents.

Antifungal resistance is associated with poorer clinical outcomes [35]. Studies have shown better clinical outcomes in patients with candidemia, and mucosal candidiasis due to *C. albicans*, *C. tropicalis*, and *C. parapsilosis* isolates that are susceptible to fluconazole, as compared to those with fluconazole-resistant isolates (92% success rate for 550 events for which the fluconazole minimum inhibitory concentration (MIC) is ≤2 mg/L, 83% success among 52 events for which the MIC is 4 mg/mL, and 37% success among 212 events for which the MIC is 8 mg/L) [35]. Furthermore, Baddley et al. [36] reported lower mortality in patients with candidemia caused by fluconazole-susceptible isolates as compared with those due to fluconazole-resistant strains (25.4% vs. 60%).

### 3.2. Biofilm Formation

As highlighted by Rodrigues et al. [1], biofilm formation by *Candida* sp. is a significant factor that hampers effective treatment of fungal infections. Most of the infections caused by *Candida* sp. are the result of biofilm formation. *Candida* sp. can form biofilm on various surfaces, including Hickman catheters, soft contact lenses, urethral stents, and corneas [37]. Biofilms provide the *Candida* sp. refuge to tolerate high concentrations of antifungals [38]. Biofilm infections are, therefore, resistant to antifungal agents and more problematic to treat than infections with planktonic cells [39]. In a case-control study to ascertain the predictors of persistent candidemia and investigate the effect of biofilm formation by *Candida* sp. isolates in adult patients with candidemia, Li et al. [40] found that biofilm formation by *C. albicans*, *C. tropicalis*, and *C. glabrata* strains was significantly higher in patients with persistent candidemia than in the controls. They also demonstrated that infection with higher biofilm-forming strains of *Candida* sp. is associated with persistent candidemia (Adjusted odds ratio, 8.03; 95% confidence interval, 2.50–25.81; *p* < 0.01).

Chandra et al. [41] reported that *C. albicans* biofilms formed on denture acrylics in vitro displayed resistance to amphotericin B, nystatin, and fluconazole. On the contrary, the same isolate grown planktonically were susceptible to these agents. El-Azizi et al. [42] assessed the effect of amphotericin B and voriconazole against the biofilm and the biofilm-dispersed cells of *C. albicans* using an in vitro biofilm model. The MIC of voriconazole and amphotericin B against the tested isolate was 0.0325 and 0.25 mg/L, respectively. Five doses of amphotericin B or voriconazole were delivered to 2-, 5-, and 10-day-old biofilms at initial concentrations of 2 and 3 m/L, respectively. The study showed that voriconazole or amphotericin B were unable to eradicate *C. albicans* biofilm, stop cell dispersion from the biofilm or cease the progression of resistance to the antifungal agents.

Paradoxical effect of echinocandins, such as caspofungin, on *Candida* sp. biofilms have also been documented [43,44]. Paradoxical growth is defined as regrowth in the presence of drug concentrations above the MIC [43]. It has been demonstrated in in vitro studies that paradoxical growth occurred more frequently in *C. albicans*, *C. parapsilosis*, *C. orthopsilosis*, and *C. metapsilosis* isolates or *C. tropicalis* when they were grown as biofilms than when they were grown planktonically [43,44].

## 4. Application of PK/PD Concepts in Optimisation of Antifungal Dosing

Unfortunately, studies exploring the PK and PD of antifungals for the treatment of *Candida* sp. infections in patients with DM are limited. However, the potential for PK changes of antimicrobials is clear. The physiological changes that drive PK/PD alteration in other patient populations e.g., critically ill patients in intensive care units and hemato-oncological patients with neutropenia are also present in patients with DM as highlighted above. In that context, to illustrate the potential of PK/PD application in the optimisation of antifungal dosing regimens, studies involving critically ill patient populations are discussed here.

Considering the obstacles of providing effective treatment for *Candida* sp. infection in diabetic patients as mentioned above, applying PK/PD concepts to make optimum antifungal dosing should be deemed fundamental. PK/PD optimised antifungal dosing regimens are likely needed to ensure a successful treatment outcome while minimising side effects and the emergence of resistance. Table 1 summarises several PK/PD studies evaluating the dosing regimens of antifungals used for *Candida* sp. infections in critically ill patients.

Mouton et al. [45] described the area under the concentration–time curve for the unbound drug fraction/MIC ratio (*f*AUC/MIC) as the PK/PD index that is associated with the efficacy of fluconazole. Based on their in-silico study of fluconazole, the authors demonstrated that infections caused by *Candida* sp. with MICs up to 2 mg/L have a high probability of cure if an *f*AUC/MIC target value of 100 is applied. They also concluded that the treatment of infections caused by *Candida* sp. can be optimised, according to the MIC of the strains.

In a population pharmacokinetic study of fluconazole in critically ill nonobese, obese, and morbidly obese patients by Alobaid et al. [46], the authors revealed that a fluconazole dose of 200 mg daily administered intravenously was insufficient to achieve an *f*AUC/MIC of 100 for strains with MICs of ≥2 mg/L in patients with BMI of >30 kg/m^2^. Therefore, a weight-based dosing regimen is more prudent in achieving the PK/PD index target. They found that an intravenous fluconazole loading dose of 12 mg/kg and maintenance dose of 6 mg/kg/day achieved PK/PD target for strains with higher MICs.

In an observational study by Ghanem-Zoubi et al. [47] which looked at the association between the AUC/MIC of fluconazole and clinical outcomes in patients with candidemia, the authors discovered that higher doses of fluconazole are needed to treat *Candida* sp. with high MICs. For *C. glabrata*, a higher AUC/MIC was observed among 30-day survivors with a median (interquartile range) of 230 (77, 539) compared to 96 (75, 164) in non-survivors. (*p* = 0.008), in parallel with a trend for lower MIC values, median 7 (1, 2) vs. 16 (8, 24) mg/L (*p* = 0.06), respectively.

Treatment of fungal infection in patients on renal replacement therapy has also been challenging. Pérez-Pitarch et al. [48] showed in their study that the approved regimen of caspofungin is inadequate to obtain the PK/PD targets (targeted AUC/MIC ratios were 865 for *C. albicans*, 450 for *C. glabrata* and 1185 for *C. parapsilosis*) in critically ill patients on hemodiafiltration. The approved regimen (loading dose of 70 mg and a daily maintenance dose of 50 mg in patients with body weight ≤80 kg or 70 mg when body weight is higher than 80 kg) was only able to achieve 16.8% and 54.1% probability of target attainment against the PK/PD target for *C. parapsilosis*, and *C. albicans* isolates, respectively, with MIC ≥ 0.1 mg/L, as demonstrated by their in silico analysis.

## 5. Conclusions

Taken altogether, existing evidence suggests that disease-driven changes in the PK/PD of antifungals can occur in DM patients affecting the ability of conventional dosing regimens to ensure effective therapy. It is, therefore, important to apply PK/PD guided dose optimisation strategies for maximising patient outcomes. However, primary data available directly from DM patients is limited to enable robust PK/PD analysis and the design optimal regimens. Therefore, further studies on antifungal PK/PD in DM patients with fungal infections are urgently need.

## Figures and Tables

**Table 1:** PK/PD studies evaluating the dosing regimens of antifungals used for *Candida* sp. infections.

Reference	Antifungal Tested	Study Design	Patient Population	Dosing Regimens Tested/Simulated	PK/PD Target	Finding
Martial (2017) [49]	Micafungin	PK study, in silico	ICU, critically ill	(I) 100 mg QD for 14 days and (II) 100 mg QD the first 4 days with 200 mg QD from day 5 (labelled indication for non-responders); alternative regimens included (III) 200 mg loading dose on day 1 followed by 100 mg QD from day 2, (IV) 200 mg loading dose followed by 150 mg QD from day 2 and (V) 200 mg QD.	AUC/MIC >3000	PTA was >91% on day 14 for MIC 0.016 mg/L for all of the dosing regimens but decreased to (I) 44%, (II) 91%, (III) 44%, (IV) 78% and (V) 91% for MIC 0.032 mg/L.
					AUC/MIC >5000	PTA varied between 62 and 96% on day 14 for MIC 0.016 mg/L.
Jullien (2017) [50]	Micafungin	PK study, in silico	ICU, critically ill	100, 150, 200, 250 and 300 mg QD	AUC/MIC >5000	For MICs ≥ 0.015–0.016 mg/L, high PTA necessitated doses between 150 and 300 mg.
Grau (2015) [51]	Micafungin	PK study, in silico	ICU, critically ill	100, 150 and 200 mg QD	AUC/MIC >285 or >3000	≥90% PTA can be achieved for *Candida spp*. and *C. parapsilosis* MICs of 0.008–0.016 and 0.125–0.25 mg/L, respectively for all dosing regimens.
Pérez Civantos (2019) [52]	Anidulafungin	PK study	ICU, critically ill, peritonitis	200 mg on day 1, then 100 mg QD	-	Anidulafungin at conventional doses reaches peritoneal fluid concentrations that exceed the minimum inhibitory concentration of the usual *Candida spp*.
García-de-Lorenzo (2016) [53]	Micafungin	PK study, in silico	ICU, critically ill, burns	100, 150 and 200 mg QD	AUC/MIC >285 or >3000	Micafungin at 100 mg/day achieved the PTA for MIC values of ≤0.008 mg/L and ≤0.064 mg/L for *non-parapsilosis Candida spp*. and *C. parapsilosis* isolates, respectively
Sinnollareddy (2015) [54]	Fluconazole	PK study, in silico	ICU, critically ill	400 mg QD	AUC/MIC >100	PTA was 92% and 67% in plasma and subcutaneous-tissue interstitial fluid, respectively, for *C. albican* isolates with MIC of 1 mg/L. Higher doses is required in order to achieve the PK/PD target in the subcutaneous-tissue interstitial fluid.
Bergner (2006) [55]	Fluconazole	PK study	ICU, critically ill, CVVHF	800 mg QD	-	A dosage of 800 mg/day is required in patients receiving CVVHF to achieve Cmax of 16 to 32 mg/L.

PK, pharmacokinetics; PD, pharmacodynamics; ICU, intensive care unit; QD, once daily; CVVHF, continuous venovenous hemofiltration; AUC/MIC, area under the concentration-time curve/minimum inhibitory concentration ratio; MIC, minimum inhibitory concentration; PTA, probability of target attainment.
